# Effects of audio and visual distraction on patients’ vital signs and tolerance during esophagogastroduodenoscopy: a randomized controlled trial

**DOI:** 10.1186/s12876-020-01274-3

**Published:** 2020-04-21

**Authors:** Masahiro Sogabe, Toshiya Okahisa, Akira Fukuya, Kaizo Kagemoto, Yasuyuki Okada, Yuka Adachi, Takeshi Kurihara, Toru Nii, Satoshi Teramae, Hironori Tanaka, Tetsu Tomonari, Koichi Okamoto, Hiroshi Miyamoto, Masahiko Nakasono, Tetsuji Takayama

**Affiliations:** 1 Department of Gastroenterology and Oncology, Tokushima University Graduate School of Biomedical Sciences, 3-18-15 Kuramoto-cho, Tokushima city, Tokushima, 770-8503 Japan; 2 Department of Internal Medicine, Shikoku Central Hospital of the Mutual aid Association of Public School teachers, Shikokuchuo, Japan; 3 Department of Internal Medicine, Tsurugi Municipal Handa Hospital, Tsurugi, Japan

**Keywords:** Esophagogastroduodenoscopy, Vital signs, Heart rate variability, Distraction, Subjective and objective assessment

## Abstract

**Background:**

Esophagogastroduodenoscopy (EGD) provides an indispensable and unambiguous inspection allowing the discovery upper gastrointestinal lesions. However, many patients are anxious about undergoing EGD. Few studies have investigated the influence on patients’ vital signs and tolerance during EGD using subjective and objective assessments. This study was a prospective randomized controlled study that investigated the influence of audio and visual distraction on EGD.

**Methods:**

We randomly divided 289 subjects who underwent EGD into 4 groups (control group, audio group, visual group, combination group) and examined their vital signs, heart rate variability (HRV), psychological items, and acceptance of distraction.

**Results:**

Pulse rate (PR) at post-distraction and post-EGD in the 3 distraction groups were significantly lower than those of control group (*p* <  0.001 and *p* <  0.01, respectively). Blood pressure (BP) during and post-EGD was significantly higher than that at pre-EGD in control group (*p* <  0.05), but no significant elevation of BP was observed during the latter half of EGD and post-EGD in the 3 distraction groups. BP at post-distraction improved significantly compared to pre-distraction in the 3 distraction groups (*p* <  0.05). There was a significant difference in the low-frequency (LF) power/ high-frequency (HF) power at post-distraction and post-EGD among the 4 groups (*p* <  0.001 and *p* <  0.001, respectively). The LF power/HF power at post-distraction and post-EGD in the 3 distraction groups was significantly lower than that in control group (*p* <  0.05). Several items of profile of mood states (POMS) and the impression of EGD at post-distraction improved significantly compared to those at pre-distraction among the 3 distraction groups (*p* <  0.05). Visual analog scale (VAS) of willingness for the next use of distraction in the 3 distraction groups was excellent because VAS was more than 70.

**Conclusions:**

Distractions effectively improved psychological factors, vital signs and some of HRV at pre and post-EGD. Distractions may suppress BP elevation during the latter half of EGD and lead to stability of HRV on EGD.

**Trial registration:**

This prospective trial was registered in the University Hospital Medical Information Network (UMIN) Clinical Trials Registry as UMIN000029637. Registered on 20 October 2017.

## Background

Medical opportunities for the use of esophagogastroduodenoscopy (EGD) for the diagnosis of and therapy for etiology of gastrointestinal complaints and upper gastrointestinal cancer have increased. The development of smaller endoscope diameters reduced the unpleasant feeling and pain during EGD, but some person avoid undergoing EGD because of strong anxiety prior to the procedure [1–3]. Sedation increases the success rate of endoscopy and patient satisfaction during the endoscopic procedure [4–8], but sedation may increase the likelihood of complications, such as hypotension and respiratory depression [9–13]. Therefore, methods to improve patient anxiety during endoscopic examinations without sedation were examined. Several noninvasive intervention techniques, such as distraction using audio, visual, and olfactory stimulation, were introduced to decrease pain and anxiety during endoscopic examinations. Listening to music or watching images during various endoscopic procedures was an effective distraction in several reports, but most these reports used subjective assessments, such as pain, anxiety, and satisfaction [1, 14–16]. Few reports investigated the efficacy of the distraction of listening to music or watching images during EGD using a combination of subjective and objective assessments, including cardiovascular responses, heart rate variability (HRV), and a psychological questionnaire. We performed a prospective single-blind randomized controlled trial to assess the influence of distractions, such as audio and visual stimuli, during EGD.

## Methods

### Study design

This study protocol is included in additional Figure 1. This study was designed as a prospective, single-blinded randomized controlled trial, and it was performed at Shikoku Central Hospital of the Mutual Aid Association of Public School teachers. The Ethics Committee in Shikoku Central Hospital of the Mutual Aid Association of Public School teachers approved the study protocol, which was registered in the University Hospital Medical Information Network (UMIN Clinical Trials Registry, number UMIN000029637).

### Diagram of procedures and subject selection

**Fig. 1:**
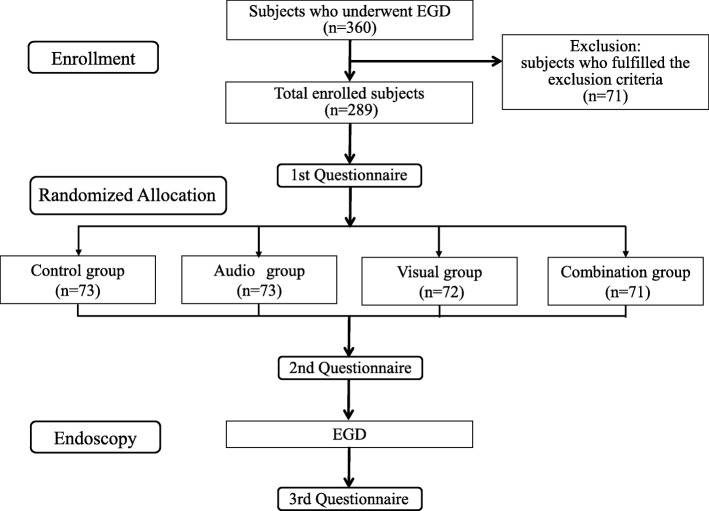
CONSORT Diagram Showing the Flow of Participants through the Trial. EGD, esophagogastroduodenoscopy

Figure 1 shows a flow diagram of the enrollment and procedures of this study. A total of 360 subjects were scheduled to receive EGD at a regular health check-up at our hospital participated in this study between October 2017 and March 2018. The study design was explained, and all subjects provided written informed consent. Seventy-one subjects were excluded from the study if they met any of the following criteria: (1) current medication use; (2) a history of severe heart failure, renal failure, hepatic failure, or chronic obstructive pulmonary disease; (3) previous abdominal surgery, including endoscopic mucosal resection (EMR) and endoscopic submucosal dissection (ESD); (4) audio or visual disability; (5) previous experience of bad feelings from audio or visual stimuli; (6) a history of anxiety or psychiatric disorders; (7) pregnant or a possibility of pregnancy; and (8) receiving a diagnosis of gastrointestinal cancer or required biopsy. Subjects presented to the endoscopy floor in the morning after a longer than 12-h fasting period. Subjects who was divided into two categories those underwent EGD for the first time and those experienced EGD previously and each subject was randomly divided into 4 groups using a sealed opaque numbered envelope method by the endoscopy nurse who assisted at EGD. All subjects sat on a sofa and rested quietly for 5 min in a private room near the endoscopy room. Subjects in control group continued to sit on the sofa and rest quietly for 10 min prior to EGD. Subjects in audio group sat on the sofa and listened to music for 10 min. Subjects in visual group sat on the sofa and watched a silent natural image for 10 min. Subjects in combination group sat on the sofa and watched a natural image while listening to music for 10 min. The study used healing music, such as country and classical music, based on the tone of a music box, which was chosen as good by 20 volunteers in a pre-meeting prior to the start of this study. The moving images used in this study were various natural images, including a mountain, forest, river, waterfall, lake, and sunset. Music and natural images were delivered using a wall-type Hi-vision liquid crystal television (TH-42AS650; Panasonic Corporation, Osaka, Japan). Pharyngeal anesthesia with lidocaine pump spray (Xylocaine Pump Spray 8%; AstraZeneca, Osaka, Japan) without any sedative agents was applied, and 5 endoscopy specialists with greater than 5 years of experience in endoscopy performed a standard EGD, including observations of the esophagus, stomach, and duodenum, using a conventional single channel endoscope (GIF-H260; Olympus, Tokyo, Japan) without knowledge of the group of the subject. The profile of mood states (POMS) and the visual analog scale (VAS) of impressions for EGD were performed at pre- and post-distraction. VAS of the acceptance of distraction was performed after EGD.

### Measurement of vital signs

Pulse rate (PR) and blood pressure (BP) were measured in the right upper arm, and peripheral blood oxygen saturation (SpO_2_) was measured at the left finger using a monitor unit (BSM-7100 Life Scope; NIHON KOHDEN CORPORATION, Tokyo, Japan). These parameters were measured 5 and 15 min after sitting on the sofa, during EGD, and 5 min after EGD procedure. Parameters during EGD were measured just after insertion of the endoscope through the esophagogastric junction (approximately 2 min from the start of EGD) and just after moving the endoscope from the stomach to the esophagogastric junction (approximately 5–7 min from the start of EGD).

### Assessment of HRV

We assessed autonomic nervous function from pre-EGD to post-EGD using power spectral analysis (PSA). HRV was measured using a Heart Rhythm Scanner (HRV analysis system from Biocom Technologies, Ark Trading Pacific, Inc.) equipped with software that performed algorithms for short-term HRV analysis. A Biocom HRS-08 Bluetooth Wireless Pulse Wave Sensor photoplethysmography monitor was used in this study, and it was clipped to the right earlobe. Data of the average R-R intervals for 5 min were subjected to PSA using the software of the HRV analysis system. The amplitudes of the low-frequency (LF) range (LF, 0.04–0.15 Hz) and high-frequency (HF) range (HF, 0.15–0.40 Hz) were analyzed using complex demodulation. These LF and HF values were designated as the LF power and HF power, respectively. HF power is fluctuation in the heart rate caused by respiration, which is mediated by cardiac parasympathetic nervous activity [17, 18]. The ratio of LF power to HF power is an index of sympathetic nervous activity [19–22]. The HF power data were converted to a logarithmic scale to analyze using linear regression in the present study.

### Psychological assessment and acceptance of distraction

We used the POMS2 questionnaire for psychological assessments between pre- and post-distraction because POMS is a self-report measure that quickly assesses transient, fluctuating feelings and enduring affective states [23, 24]. The POMS2 is composed of 35 items and 8 subscale scores. We also used the VAS, which consists of a 100-mm horizontal line scored from 0 to 100 to rate the degrees of strain, anxiety, and fear of EGD. All subjects answered the POMS and provided their impressions of EGD immediately after sitting on the sofa and 15 min after sitting on the sofa, but prior to EGD. After EGD, subject used the VAS to rate their degrees of satisfaction, usefulness, and willingness to assess the subject’s acceptance of distraction.

### Outcomes

The primary outcome measures were psychological factors including POMS and impression for EGD at post-distraction and acceptance of distraction including degrees of satisfaction, usefulness, and willingness for the next use at post EGD. The secondary outcome measures were vital signs, HRV at post-distraction, during EGD, and at post-EGD. Vital signs and HRV were measured at pre-EGD (5 min after sitting on the sofa), pre-EGD (15 min after sitting on the sofa), during the early and the latter half of EGD, and 5 min after the end of EGD.

### Statistical analysis

We assumed that the appropriate sample size for the randomized subjects was over 180 subjects based on the requirement of a significant difference between 4 groups with a significance level of 0.05, power of 80%, and, effect size of 0.25. Additionally, the rate of subjects who fill exclusion criteria or who received a diagnosis of gastrointestinal cancer or a biopsy was 30–40% by referring to our previous prospective randomized trial on endoscopy. Therefore, the planned required number of subject who receive EGD was over 300. Quantitative data, including subject characteristics, vital signs, POMS score, and VAS scores of impressions for EGD and acceptance of distraction, are expressed as the means ± standard deviation (SD). Parameters of autonomic nervous function are expressed as the means ± standard error of the mean (SEM). All significant differences at a *P* value less than 0.05 were considered significant. The χ^2^-test or Mann-Whitney U-test was used for comparisons between 2 groups or pre- and post-distraction in same group. The m × n χ^2^-test or Kruskal Wallis test was used to analyze differences among 3 or 4 groups. If the Kruskal Wallis test revealed differences between the groups, then post-hoc pairwise comparisons were performed using the Mann-Whitney U test with Bonferroni correction. All analyses were performed using Med Calc Software (Broekstraat, Mariakerke, Belgium).

## Results

### Baseline characteristics of subjects

Table 1 shows the baseline characteristics in the 4 groups. There was no significant difference in age, gender, smoking, drinking, experience or duration of EGD, POMS, and the impression for EGD among the 4 groups.

**Table 1:** Baseline characteristics of subjects in the four groups

	Total subjects (***n*** = 289)	Control group (***n*** = 73)	Audio group (***n*** = 73)	Visual group (***n*** = 72)	Combination group (***n*** = 71)	***P***-value
**Age (years)**	52.1 ± 6.7	52.8 ± 6.7	52.5 ± 6.6	50.7 ± 7.3	52.3 ± 6.2	NS
**Gender**						
**Male**	171	46	45	40	40	NS
**Female**	118	27	28	32	31	
**Smoking (+/−)**	35/254	10/63	9/64	12/60	4/67	NS
**Drinking (+/−)**	165/124	45/28	42/31	40/32	38/33	NS
**Number of EGD** **experience**	4.3 ± 3.4 (0–20)	4.4 ± 3.9 (0–20)	4.5 ± 3.8 (0–16)	4.1 ± 2.6 (0–11)	4.4 ± 3.3 (0–15)	NS
**Duration of EGD (sec)**	341 ± 98	330 ± 96	351 ± 96	355 ± 116	329 ± 81	NS
**First score of POMS**						
**(negative mood)**						
**A-H**	46.5 ± 7.5	46.8 ± 8.0	46.2 ± 6.9	46.6 ± 6.5	46.3 ± 8.3	NS
**C-B**	48.9 ± 8.0	48.4 ± 8.5	49.4 ± 8.1	49.6 ± 7.2	48.3 ± 8.2	NS
**D-D**	48.9 ± 7.2	49.0 ± 7.5	49.8 ± 8.2	48.5 ± 6.7	48.5 ± 6.3	NS
**F-I**	45.7 ± 8.2	46.0 ± 9.6	46.2 ± 7.1	45.3 ± 7.1	45.5 ± 9.0	NS
**T-A**	52.3 ± 9.8	53.0 ± 10.9	50.9 ± 8.6	54.1 ± 9.0	51.4 ± 10.5	NS
**TMD**	47.3 ± 7.8	48.0 ± 9.0	47.5 ± 7.6	47.6 ± 6.7	46.2 ± 7.6	NS
**(positive mood)**						
**V-V**	54.6 ± 9.5	54.4 ± 10.6	55.0 ± 8.8	55.3 ± 9.1	53.5 ± 9.3	NS
**F**	58.6 ± 8.9	56.8 ± 9.7	58.6 ± 8.0	59.2 ± 8.7	59.8 ± 9.2	NS
**VAS of impression** **for EGD**						
**Strain**	48.7 ± 27.9	46.6 ± 26.7	47.1 ± 26.9	53.7 ± 27.9	47.3 ± 30.2	NS
**Anxiety**	40.3 ± 27.7	38.5 ± 28.1	44.5 ± 26.8	39.5 ± 25.8	38.8 ± 30.0	NS
**Fear**	32.5 ± 26.7	29.6 ± 25.3	36.3 ± 28.5	32.3 ± 23.2	31.8 ± 29.6	NS

*A-H* Anger-hostility; *C-B* Confusion-bewilderment; *D-D* Depression-dejection; *EGD* Esophagogastroduodenoscopy; *F* friendship; *F-I* Fatigue-languid; *POMS* Profile of mood states; *T-A* Tension-anxiety; *TMD* Total mood distress; *VAS* Visual analog scale; *V-V* Vigor-vitality

Data represent the means ± standard deviation (SD) and number for categorical variables. The *P*-value is based on the m × n χ^2^ test or Kruskal Wallis test. Significance is at the 5% level

### Change in vital signs

**Fig. 2:**
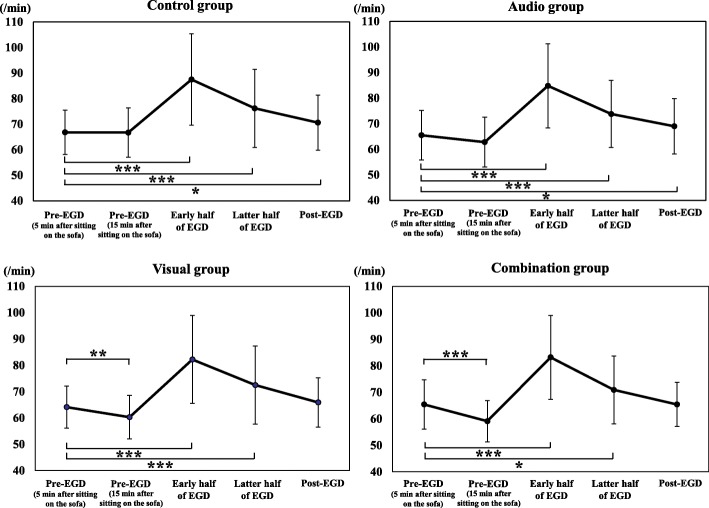
Changes in pulse rate from pre-distraction to post-EGD in the 4 groups. EGD, esophagogastroduodenoscopy; *p < 0.05; **p < 0.005; ***p < 0.001

Figure 2 shows the changes in PR from pre-EGD to post-EGD in the 4 groups. PRs during the early and the latter half of EGD, and 5 min after the end of EGD were significantly higher than pre-EGD (5 min after sitting on the sofa) in control group and audio group (*p* <  0.001, *p* <  0.001, and *p* <  0.05). PRs during the early and the latter half of EGD were significantly higher than pre-EGD (5 min after sitting on the sofa) in visual group (*p* <  0.001 and *p* <  0.005) and combination group (*p* <  0.001 and *p* <  0.05), but there was no significant difference in PR between pre-EGD (5 min after sitting on the sofa) and 5 min after the end of EGD, and PR at pre-EGD (15 min after sitting on the sofa) was significantly lower than pre-EGD (5 min after sitting on the sofa) in visual group (*p* <  0.005) and combination group (*p* <  0.001).

**Fig. 3:**
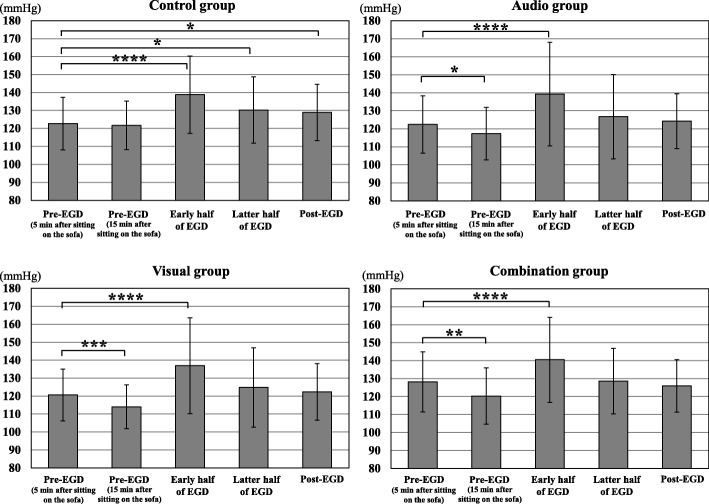
Changes in blood pressure from pre-distraction to post-EGD in the 4 groups. EGD, esophagogastroduodenoscopy; *p < 0.05; **p < 0.01; ***p < 0.005; ****p < 0.001

Figure 3 shows the changes in BP from pre-EGD to post-EGD in the 4 groups. BPs during the early and the latter half of EGD, and 5 min after the end of EGD were significantly higher than pre-EGD (5 min after sitting on the sofa) in control group (*p* <  0.001, *p* <  0.05, and *p* <  0.05). BP during the early half of EGD was significantly higher than pre-EGD (5 min after sitting on the sofa) in the 3 distraction groups (*p* <  0.001), but no significant BP elevation was observed during the latter half of EGD and 5 min after the end of EGD. BP at pre-EGD (15 min after sitting on the sofa) was significantly lower than pre-EGD (5 min after sitting on the sofa) in the 3 distraction groups (*p* <  0.05).

**Fig. 4:**
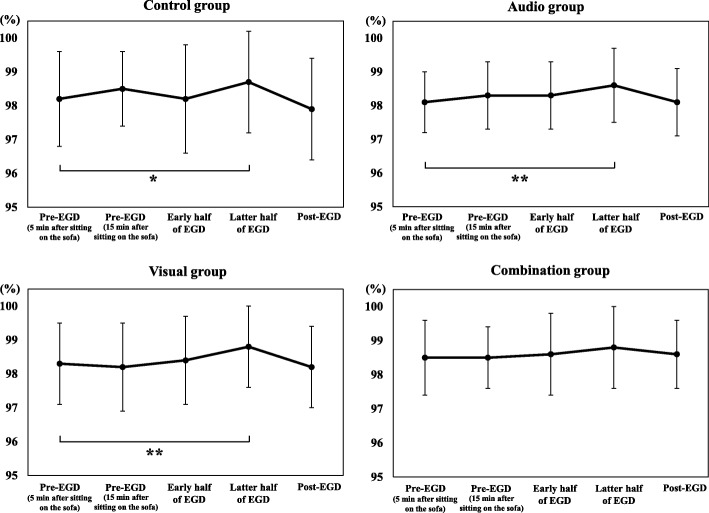
Changes in blood oxygen saturation from pre-distraction to post-EGD in the 4 groups. EGD, esophagogastroduodenoscopy; *p < 0.01; **p < 0.005

Fig. 4 shows the changes in SpO_2_ from pre-EGD to post-EGD in the 4 groups. SpO_2_ during the latter half of EGD was significantly higher than pre-EGD (5 min after sitting on the sofa) in control group, audio group, and visual group (*p* <  0.01, *p* <  0.005, and *p* <  0.005).

### Comparison of PR at each point

Table 2 shows a comparison of PR at each point among the 4 groups. There was no significant difference in PR at pre-EGD (5 min after sitting on the sofa), during the early half of EGD, and the latter half of EGD among the 4 groups. However, there was a significant difference in PR at pre-EGD (15 min after sitting on the sofa) and 5 min after the end of EGD among the 4 groups on the Kruskal Wallis test (*p* <  0.001 and *p* <  0.01). Post-hoc pairwise comparisons revealed that PRs at pre-EGD (15 min after sitting on the sofa) and 5 min after the end of EGD in the 3 distraction groups were significantly lower than control group.

**Table 2:** Comparison of pulse rate at four points among the four groups

		Control group	Audio group	Visual group	Combination group	*P*-value
Pre-EGD (5 min after sitting on the sofa) (/min)	Mean	66.8	65.5	64.1	65.4	NS
	SD	8.7	9.7	8.0	9.3	
	Max	49	45	44	42	
	Min	93	92	87	87	
Pre-EGD (15 min after sitting on the sofa) (/min)	Mean	66.7a	62.8b	60.3bc	59.1c	< 0.001
	SD	9.7	9.8	8.3	7.8	
	Max	95	94	85	80	
	Min	47	43	42	40	
Early half of EGD (/min)	Mean	87.5	84.8	82.2	83.2	NS
	SD	17.9	16.4	16.7	15.8	
	Max	136	120	135	120	
	Min	56	51	46	55	
Latter half of EGD (/min)	Mean	76.2	73.8	72.5	70.9	NS
	SD	15.3	13.1	14.8	12.8	
	Max	126	105	126	107	
	Min	46	42	42	41	
5 min after the end of EGD (/min)	Mean	70.6a	69.0b	65.9bc	65.4c	< 0.01
	SD	10.8	10.8	9.4	8.3	
	Max	103	95	90	85	
	Min	50	45	44	40	

*EGD* Esophagogastroduodenoscopy; *SD* Standard deviation

The *P*-value is based on the Kruskal Wallis test. Significance is at the 5% level. Post hoc pairwise comparisons were performed using the Mann-Whitney U test with Bonferroni correction. Different letters indicate a significant difference at the 0.00833 (0.05/6) level

### Comparison of BP at each point

Table 3 shows a comparison of BP at each point among the 4 groups. There was no significant difference in BP at pre-EGD (5 min after sitting on the sofa), during the early half of EGD, and the latter half of EGD among the 4 groups. However, there was a significant difference in BP at pre-EGD (15 min after sitting on the sofa) and 5 min after the end of EGD among the 4 groups on the Kruskal Wallis test (*p* <  0.005 and *p* <  0.05). Post-hoc pairwise comparisons revealed that BP at pre-EGD (15 min after sitting on the sofa) and 5 min after the end of EGD in visual group was significantly lower than control group.

**Table 3:** Comparison of blood pressure at four points among the four groups

		Control group	Audio group	Visual group	Combination group	*P*-value
Pre-EGD (5 min after sitting on the sofa) (mmHg)	Mean	122.8	122.4	120.6	128.3	NS
	SD	14.6	15.9	14.4	16.7	
	Max	157	161	157	178	
	Min	96	90	91	97	
Pre-EGD (15 min after sitting on the sofa) (mmHg)	Mean	121.7b	117.3	114.0a	120.3b	< 0.005
	SD	13.5	14.6	12.2	15.6	
	Max	158	156	158	171	
	Min	98	90	89	90	
Early half of EGD (mmHg)	Mean	138.8	139.3	136.9	140.5	NS
	SD	21.5	28.8	26.7	23.7	
	Max	188	214	224	200	
	Min	91	88	87	79	
Latter half of EGD (mmHg)	Mean	130.3	126.7	124.8	128.6	NS
	SD	18.5	23.4	22.1	18.3	
	Max	180	195	194	171	
	Min	93	88	90	78	
5 min after the end of EGD (mmHg)	Mean	128.9a	124.2	122.3b	126.0	< 0.05
	SD	15.7	15.2	15.7	14.6	
	Max	193	164	182	166	
	Min	97	92	96	100	

*EGD* Esophagogastroduodenoscopy; *SD* Standard deviation

The *P*-value is based on the Kruskal Wallis test. Significance is at the 5% level. Post hoc pairwise comparisons were performed using the Mann-Whitney U test with Bonferroni correction. Different letters indicate a significant difference at the 0.00833 (0.05/6) level

### Comparison of SpO_2_ at each point

Table 4 shows a comparison of SpO_2_ at each point among the 4 groups. There was no significant difference in SpO_2_ at pre-EGD (5 min after sitting on the sofa), pre-EGD (15 min after sitting on the sofa), during the early half of EGD, and the latter half of EGD among the 4 groups. However, there was a significant difference in SpO_2_ at 5 min after the end of EGD among the 4 groups on the Kruskal Wallis test (*p* <  0.01). Post-hoc pairwise comparisons revealed that SpO_2_ at 5 min after the end of EGD in combination group were significantly higher than control group.

**Table 4:** Comparison of blood oxygen saturation at four points among the four groups

		Control group	Audio group	Visual group	Combination group	*P*-value
Pre-EGD (5 min after sitting on the sofa) (%)	Mean	98.2	98.1	98.3	98.5	NS
	SD	1.4	0.9	1.2	1.1	
	Max	100	100	100	100	
	Min	93	95	94	96	
Pre-EGD (15 min after sitting on the sofa) (%)	Mean	98.5	98.3	98.2	98.5	NS
	SD	1.1	1.0	1.3	0.9	
	Max	100	100	100	100	
	Min	95	96	94	96	
Early half of EGD (%)	Mean	98.2	98.3	98.4	98.6	NS
	SD	1.6	1.0	1.3	1.2	
	Max	100	100	100	100	
	Min	91	95	93	95	
Latter half of EGD (%)	Mean	98.7	98.6	98.8	98.8	NS
	SD	1.5	1.1	1.2	1.2	
	Max	100	100	100	100	
	Min	92	96	95	95	
5 min after the end of EGD (%)	Mean	97.9b	98.1b	98.2	98.6a	< 0.01
	SD	1.5	1.0	1.2	1.0	
	Max	100	100	100	100	
	Min	94	96	95	95	

*EGD* Esophagogastroduodenoscopy; *SD* Standard deviation

The *P*-value is based on the Kruskal Wallis test. Significance is at the 5% level. Post hoc pairwise comparisons were performed using the Mann-Whitney U test with Bonferroni correction. Different letters indicate a significant difference at the 0.00833 (0.05/6) level

### Changes in HRV

**Fig. 5:**
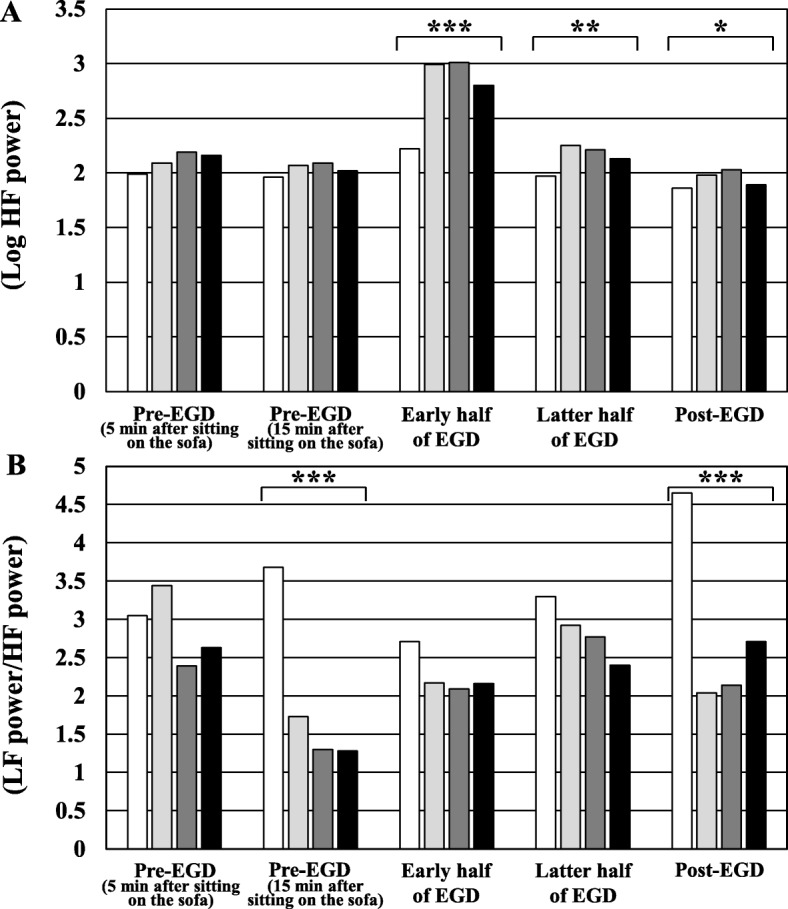
Changes in heart rate variability from pre-EGD to post-EGD in the 4 groups. **a** Comparisons of Log HF powers from pre-EGD to post-EGD in the 4 groups. **b** Comparisons of LF power/ HF power from pre-EGD to post-EGD in the 4 groups. The white bar indicates the values of control group. The light gray bar indicates values of audio group. The dark gray bar indicates values of visual group. The black bar indicates values of combination group. EGD, esophagogastroduodenoscopy; HF, high frequency; LF, low frequency; *P < 0.05; **P < 0.01; ***P < 0.001

Figure 5-A shows a comparison of Log HF power from pre-EGD to post-EGD among the 4 groups. There was a significant difference in Log HF power during the early and the latter half of EGD, and 5 min after the end of EGD among the 4 groups on the Kruskal Wallis test (*p* <  0.001, *p* <  0.01, and *p* <  0.05). Post-hoc pairwise comparisons revealed that Log HF powers during the early half of EGD in the 3 distraction groups was significantly higher than control group.

Figure 5-B shows a comparison of LF power/ HF power from pre-EGD to post-EGD between the 4 groups. There was a significant difference in LF power/ HF power at pre-EGD (15 min after sitting on the sofa) and 5 min after the end of EGD among the 4 groups on the Kruskal Wallis test (*p* <  0.001 and *p* <  0.001). Post-hoc pairwise comparisons revealed that LF power/ HF power at pre-EGD (15 min after sitting on the sofa) and 5 min after the end of EGD in the 3 distraction groups was significantly lower than control group.

### Influence of distraction on POMS and the impression for EGD

Table 5 shows a comparison of POMS and the impression for EGD between pre- and post-distraction in the 3 distraction groups. The score for negative mood at post-distraction was significantly lower than at pre-distraction in the 3 distraction groups (*p* <  0.05). The VAS scores of strain, anxiety, and fear for EGD at post-distraction were significantly lower than pre-distraction in audio group (*p* <  0.05, *p* <  0.05, and NS), visual group (*p* <  0.005, NS, and NS), and combination group (*p* <  0.001, *p* <  0.05, and *p* <  0.05).

**Table 5:** Comparison of POMS and the impression for EGD between pre- and post-distraction in the three distraction groups

Group	POMS and impression for EGD	Pre-distraction	Post-distraction	*P*-value
Audio group	(POMS: Score of negative mood)			
	A-H	46.2 ± 6.9	44.1 ± 6.9	< 0.05
	C-B	49.4 ± 8.1	46.8 ± 7.8	< 0.05
	D-D	49.8 ± 8.2	47.3 ± 7.3	< 0.05
	F-I	46.2 ± 7.1	43.2 ± 7.6	< 0.01
	T-A	50.9 ± 8.6	46.0 ± 9.2	< 0.001
	TMD	47.5 ± 7.6	43.9 ± 7.7	< 0.005
	(POMS: Score of positive mood)			
	V-V	55.0 ± 8.8	55.4 ± 9.4	NS
	F	58.6 ± 8.0	58.6 ± 9.8	NS
	(VAS of impression for EGD)			
	Strain	47.1 ± 26.9	37.5 ± 23.9	< 0.05
	Anxiety	44.5 ± 26.8	34.3 ± 25.4	< 0.05
	Fear	36.3 ± 28.5	27.3 ± 25.2	NS
Visual group	(POMS: Score of negative mood)			
	A-H	46.6 ± 6.5	44.3 ± 6.9	< 0.05
	C-B	49.6 ± 7.2	46.6 ± 7.0	< 0.005
	D-D	48.5 ± 6.7	46.0 ± 5.9	< 0.005
	F-I	45.3 ± 7.1	42.5 ± 6.4	< 0.01
	T-A	54.1 ± 9.0	47.2 ± 7.9	< 0.001
	TMD	47.6 ± 6.7	43.7 ± 6.3	< 0.001
	(POMS: Score of positive mood)			
	V-V	55.3 ± 9.1	54.4 ± 11.1	NS
	F	59.2 ± 8.7	58.6 ± 10.5	NS
	(VAS of impression for EGD)			
	Strain	53.7 ± 27.9	40.5 ± 22.0	< 0.005
	Anxiety	39.5 ± 25.8	34.9 ± 24.4	NS
	Fear	32.3 ± 23.2	31.0 ± 26.7	NS
Combination group	(POMS: Score of negative mood)			
	A-H	46.3 ± 8.3	42.6 ± 6.8	< 0.001
	C-B	48.3 ± 8.2	44.9 ± 6.7	< 0.005
	D-D	48.5 ± 6.3	45.1 ± 5.9	< 0.001
	F-I	45.5 ± 9.0	40.5 ± 6.5	< 0.001
	T-A	51.4 ± 10.5	43.9 ± 8.4	< 0.001
	TMD	46.2 ± 7.6	42.2 ± 6.6	< 0.001
	(POMS: Score of positive mood)			
	V-V	53.5 ± 9.3	53.7 ± 10.9	NS
	F	59.8 ± 9.2	59.8 ± 10.5	NS
	(VAS of impression for EGD)			
	Strain	47.3 ± 30.2	28.3 ± 25.9	< 0.001
	Anxiety	38.9 ± 30.0	27.6 ± 26.2	< 0.05
	Fear	31.8 ± 29.6	22.6 ± 26.5	< 0.05

*A-H* Anger-hostility; *C-B* Confusion-bewilderment; *D-D* Depression-dejection; *EGD* Esophagogastroduodenoscopy; *F* Friendship; *F-I* Fatigue-languid; *POMS* Profile of mood states; *T-A* Tension-anxiety; *TMD* Total mood distress; *VAS* Visual analog scale; *V-V* Vigor-vitality

Data represent the means ± standard deviation (SD). The *P*-value is based on the Mann-Whitney U-test. Significance is at the 5% level

### Acceptance of distraction after EGD

Table 6 shows a comparison of the acceptance of the distraction after EGD among the 3 distraction groups.

**Table 6:** Comparison of the acceptance of the distraction after EGD among the three distraction groups

	Audio group	Visual group	Combination group	*P*-value
Usefulness of the distraction	72.3 ± 16.5	67.7 ± 15.5_a_	76.4 ± 17.4_b_	< 0.005
Satisfaction of the distraction	68.6 ± 19.4	64.2 ± 18.6_a_	74.0 ± 18.6_b_	< 0.005
Willingness for the next use of distraction	76.4 ± 17.3	73.1 ± 20.3	78.1 ± 17.7	NS

*EGD* Esophagogastroduodenoscopy

Data represent the means ± standard deviation (SD)

The *P*-value is based on the Kruskal Wallis-test. Significance is at the 5% level. Post hoc pairwise comparisons were performed using the Mann-Whitney U test with Bonferroni correction. Different letters indicate a significant difference at the 0.01667 (0.05/3) level

There was a significant difference in usefulness of the distraction and satisfaction of the distraction among the 3 groups on the Kruskal Wallis test (*p* <  0.005). Post-hoc pairwise comparisons revealed that the usefulness of the distraction and the satisfaction of the distraction in combination group was significantly higher than visual group. Although there was no significant difference in willingness for the next use of the distraction among the 3 groups, the degree of willingness for the next use of the distraction was excellent because the VAS was more than 70 in the 3 groups.

## Discussion

Several studies examined the benefits of audio and video distraction during various endoscopy procedures [1, 14–16, 25–31]. However, no clear recommendations for distraction were established because assessments in previous reports were based solely on subjective items, such as tolerance, pain, anxiety and satisfaction. To our knowledge, this is the first study evaluating the influence of distractions on EGD using subjective and objective measures.

The incidence of gastric cancer decreased worldwide in recent decades, but it remains a major cause of cancer-related mortality [32]. Therefore, EGD is an indispensable instrument to discover upper gastrointestinal cancer and perform endoscopic treatments, such as EMR and ESD. Appropriate methods for amount and use of sedation, improved techniques and apparatus for endoscopy were established. However, serious complications related to endoscopic procedures and sedation remain and are a problematic. The exact mechanisms of these complications remain conjectural, but the importance of vital sign changes and causes related to the autonomic nerve system were demonstrated. There were several reports of an association with endoscopy procedure and the autonomic nervous system, but few reports associated EGD and distractions using objective assessments, such as vital signs and autonomic nervous function [33, 34].

The present study demonstrated that BP at post-distraction (i.e., immediately pre-EGD) in the distraction groups improved significantly, but no improvement in BP was observed in the no distraction group. BP during and post-EGD increased significantly in the no distraction group, but no significant BP elevation was observed during the latter half of EGD and post-EGD in the distraction groups. There was also no significant elevation in PR at post-EGD in visual group, and combination group. These results suggest that distraction suppresses vital sign elevation at pre-EGD, during a portion of EGD, and at post-EGD.

There was a significant difference in Log HF power during the early half of EGD between the control group and distraction groups. The vomiting reflex affects the early half of EGD because of endoscope insertion. Additionally, gastrointestinal distention by air supply from the top of endoscopy may induce activation of a vagal reflex [35, 36]. Promotion of parasympathetic nervous activity may be induced by not only the influence of distraction but also vomiting reflex and gastrointestinal distention because of stimulation of endoscope insertion.

The LF power/HF power ratios at post-distraction (i.e., immediately pre-EGD) and post-EGD in the distraction groups were significantly lower than control group. These results suggest that distraction inhibited activity of sympathetic nervous function.

The psychological influence of music or visual distraction on endoscopy procedures remains controversial, but the number of articles reporting positive effects on anxiety levels appears slightly greater than negative effects articles [1, 14–16, 25–31, 37]. The present study demonstrated that the scores for negative mood based on POMS and impression of EGD at post-distraction (immediately pre-EGD) improved significantly compared to the baseline condition in the distraction groups. Acceptance of distraction in all distraction groups was relatively good.

The present study had some limitations that should be noted. First, we used healing music and natural images that were selected as good by 20 volunteers in a pre-meeting for the selection of music prior to the start of the present study. However, whether the music and images used in this study were the more suitable for each subject is not clear. Second, different results may occur between persons who underwent EGD for the first time and persons who experienced EGD previously. Further investigation of subjects who undergo EGD for the first time or comparisons between persons who undergo EGD for the first time and repeated procedures is required. Last, there was a possibility of selection bias because all of the participants in the present study were healthy individuals undergoing a medical check-up. The mean of age of the subjects was relatively young. Whether sick persons or elderly populations would produce similar results to the present study is not clear. Further studies are necessary to resolve these limitations.

## Conclusions

The present study demonstrated that distractions effectively improved psychological factors, vital signs, and HRV at pre and post-EGD. Additionally, distractions suppressed BP elevations during the latter half of EGD and sympathetic nerve function elevation at post-EGD. Although it is important for persons to undergo EGD to discover gastrointestinal lesions, the necessity for improvements in various physical and psychological conditions at pre-EGD should be considered.

## Supplementary information

**Additional file 1: Figure 1.** This study protocol. BP, blood pressure; EGD, esophagogastroduodenoscopy; HF, high-frequency; HRV, heart rate variability; LF, low-frequency; POMS, profile of mood states; PR, pulse rate.

## Data Availability

The datasets used and analyzed during the current study will be available from the corresponding author on reasonable request.

## Abbreviations

BP: Blood pressure
EGD: Esophagogastroduodenoscopy
HF: High-frequency
HRV: Heart rate variability
LF: Low-frequency
POMS: Profile of mood states
PR: Pulse rate
PSA: Power spectral analysis
VAS: Visual analog scale
