# Supplementary material for: Effects of audio and visual distraction on patients’ vital signs and tolerance during esophagogastroduodenoscopy: a randomized controlled trial
Source: BMC Gastroenterol. 2020 Apr 21;20:122. doi: 10.1186/s12876-020-01274-3 (PMC7175521; doi:10.1186/s12876-020-01274-3)
Supplement: Supplementary file 1 — Additional file 1: Figure 1. This study protocol. BP, blood pressure; EGD, esophagogastroduodenoscopy; HF, high-frequency; HRV, heart rate variability; LF, low-frequency; POMS, profile of mood states; PR, pulse rate. [file 12876_2020_1274_MOESM1_ESM.pdf]

Protocol

Single-blind, prospective, randomized controlled trial.

Total enrolled subjects
- Control group : subjects sitting on the sofa
- Audio group : subjects sitting on the sofa while listening to music
- Visual group : subjects sitting on the sofa while watching a silent natural image
- Combination group : subjects sitting on the sofa and watching a natural image while listening to music

	(Private room) (5 mins)	(Private room) (10 mins)	(Endoscopy room) (5 ~ 7 mins)	(Private room) (5 mins)
• Control group	Rest	Rest	EGD	Rest
• Audio group	Rest	Distraction (music)	EGD	Rest
• Visual group	Rest	Distraction (image)	EGD	Rest
• Combination group	Rest	Distraction (music and image)	EGD	Rest

Outcome

The primary outcome
- Psychological factor (POMS, Impression of EGD)
- Acceptance of distraction

The secondary outcome
- Vital sign (PR, BP)
- HRV (HF power, LF power/HF power)

(Survey components)

Vital sign (PR, BP)	○	○	○	○	○
HRV (HF, LF power)	○	○	○	○	○
POMS	○	○			
Impression for EGD	○	○			
Acceptance of distraction					○
